# Amine-Modified Diatomaceous Earth Syringe Platform (DeSEI) for Efficient and Cost-Effective EV Isolation

**DOI:** 10.3390/ijms26146843

**Published:** 2025-07-16

**Authors:** Hyo Joo Lee, Jinkwan Lee, Namheon Kim, Yong Shin

**Affiliations:** 1 Department of Biotechnology, College of Life Science and Biotechnology, Yonsei University, Seoul 03722, Republic of Korea; hyoj0125@gmail.com; 2 Infusiontech, 38 Heungan-daero, 427 Beon-gil, Dongan-gu, Anyang-si 14059, Republic of Korea; jklee@infusiontech.co.kr (J.L.); nhkim@infusiontech.co.kr (N.K.)

**Keywords:** extracellular vesicle, EV isolation, nanomaterials, diatomaceous earth, dimethyl sulfide, sample preparation

## Abstract

Conventional methods for isolating extracellular vesicles (EVs) are often limited by long processing times, a low purity, and a reliance on specialized equipment. To overcome these challenges, we developed the DeSEI (amine-functionalized Diatomaceous earth-based Syringe platform for EV Isolation), a novel platform employing low-cost, amine-functionalized diatomaceous earth (ADe) within a simple syringe–filter system. The capture mechanism leverages the electrostatic interaction between the positively charged ADe and the negatively charged EV surface, enabling a rapid and efficient isolation. The optimized 30 min protocol yields intact EVs with morphology, size, and protein markers comparable to those from ultracentrifugation, ensuring minimal cellular contamination. Notably, DeSEI exhibited a nearly 60-fold higher recovery efficiency of EV-derived miRNA compared to ultracentrifugation. The platform further proved its versatility with a rapid one-step miRNA extraction protocol and a user-friendly cartridge format. The direct miRNA extraction capability is particularly advantageous for a streamlined biomarker analysis, while the cartridge design illustrates a clear pathway toward developing point-of-care diagnostic tools. The DeSEI offers a promising alternative to existing methods for EV-based research by providing a combination of speed, simplicity, and procedural flexibility that does not require specialized equipment.

## 1. Introduction

Extracellular vesicles (EVs) are key mediators of intercellular communication, participating in a wide range of physiological and pathological processes by delivering bioactive cargo to recipient cells [1,2,3,4]. They carry a diverse array of molecules—including proteins, lipids, nucleic acids, and glycans—that reflect the state and origin of their parent cells and are found abundantly in various body fluids. EVs play crucial roles not only in normal physiological processes, such as immune surveillance, inflammation, and oxidative stress, but also in the onset and progression of numerous diseases, including cancer and neurodegenerative disorders [5]. Consequently, there has been a surge of research interest in EVs, driven by their potential as biomarkers for disease diagnosis and as platforms for therapeutic delivery [6,7,8,9]. To fully understand the biological functions of EVs and to harness their clinical potential, isolation methods that minimize damage while ensuring a high purity and yield are imperative, as these factors directly impact the accuracy and reproducibility of subsequent downstream analyses [10,11].

Conventional EV isolation techniques have several technical challenges. Ultracentrifugation (UC), while often considered the “gold standard″, is time-consuming, requires specialized equipment, and has notable drawbacks. These include the potential EV damage or aggregation from high centrifugal forces and often result in a low purity and yield. Moreover, its multi-step nature demands significant technical expertise [12]. Precipitation methods, though simpler and less expensive, suffer from a very low purity due to the non-specific co-precipitation of proteins and other macromolecules, and residual reagents can interfere with subsequent analyses [13]. More recently, size exclusion chromatography (SEC) has acquired a notable degree of popularity; however, its application is constrained by the volume of the sample and the capacity of the column, often necessitating an additional concentration step [12,14]. In summary, most existing methods struggle to balance the trade-off between yield and purity and are further constrained by a low throughput, complexity, and long processing times, making them suboptimal for large-scale production or rapid diagnostics in clinical settings [9,15]. Therefore, the development of a simple and rapid technology is required to overcome these limitations [16].

To overcome the limitations of conventional EV isolation methods, various strategies have been developed. Representative examples include affinity-based techniques, which enhance specificity and purity [17], and microfluidic platforms, which are attractive for point-of-care diagnostics and high-throughput applications [18]. In this study, we employed solid-phase extraction (SPE) technology, a technique widely used in analytical chemistry that selectively separates and purifies target substances from complex mixtures based on solid–liquid phase interactions. Its advantages include simplicity, a high reproducibility, and the ability to confer selectivity by functionalizing the solid surface [19,20,21]. Recently, the development of EV isolation technologies using SPE-based platforms has gained attention as a promising alternative to conventional methods [22,23,24]. For this purpose, we selected diatomaceous earth (DE) as the solid phase. DE is characterized by its exceptionally high specific surface area, well-developed porous structure, and unique three-dimensional nanostructure [25,26,27]. Owing to these physicochemical properties, DE is already widely utilized in various industrial and research fields as an adsorbent, a filtration aid, a chromatography separation medium, and for biomaterial purification [28,29,30,31,32,33]. Furthermore, DE is biocompatible, chemically stable, environmentally friendly, and available at a low cost for large-scale production [34,35,36]. Its high adsorption capacity and fine porous structure suggest that DE possesses ideal characteristics for selectively capturing and isolating nano-sized particles like EVs [37].

Leveraging these advantageous properties of DE, we introduce a novel strategy for EV isolation centered on the use of amine-functionalized diatomaceous earth (ADe) as an efficient and low-cost solid-phase matrix. The ADe captures EVs through electrostatic interactions. To translate this principle into a practical and user-friendly format, the ADe solid support is integrated into a disposable syringe filter system. This format was chosen to ensure a simple and effective isolation of the EV-bound solid phase from the sample liquid, obviating the necessity for specialized equipment. We have termed this integrated platform ‘DeSEI’ (amine-functionalized Diatomaceous earth Syringe platform for EV Isolation). The DeSEI technology is designed to provide a truly streamlined solution, enabling the simple, rapid, and effective isolation of EVs. Ultimately, this approach significantly reduces the dependency on specialized laboratory equipment and ensures a high reproducibility across experiments, addressing key limitations of current techniques.

## 2. Results

### 2.1. The Principle and Workflow of the DeSEI

**Figure 1:**
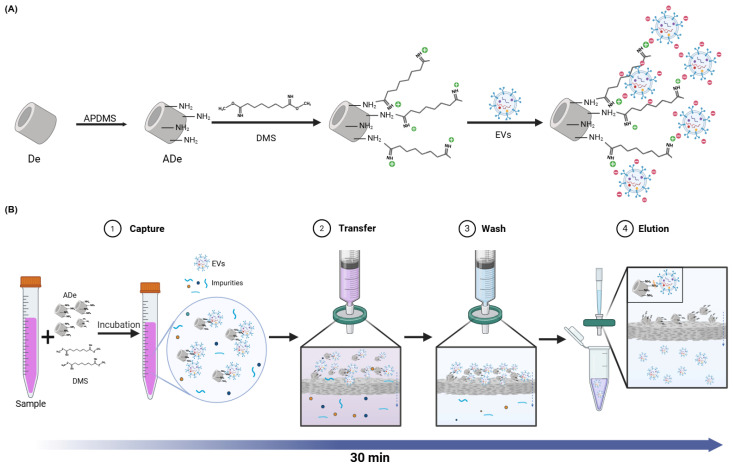
A schematic of the principle and the isolation of extracellular endosomes using the DeSEI. (**A**) A schematic of the capture mechanism. The surface of the DE is functionalized with APDMS to introduce amine groups, creating ADe. DMS then acts as a cross-linker, enabling the capture of negatively charged EVs onto the positively charged ADe surface through electrostatic interactions. (**B**) The DeSEI workflow. First, ADe and DMS are added to the sample and incubated to facilitate EV capture. Next, the EV-bound ADe is isolated, and debris is removed using a syringe filter. Finally, an elution buffer is used to disrupt the electrostatic bonds, releasing the purified EVs from the ADe. The entire four-step process is completed in under 30 min. (This figure was created with BioRender.com). Abbreviations: DeSEI, amine-functionalized Diatomaceous earth Syringe platform for EV Isolation; ADe, Amine-functionalized Diatomaceous earth; DE, Diatomaceous earth; and EVs, extracellular vesicles.

The underlying principle of the EV capture using the DeSEI is illustrated in Figure 1A. First, ADe was prepared by modifying the surface of the DE with 3-aminopropyl (diethoxy) methylsilane (APDMS) to introduce amine groups. To further enhance the EV binding efficiency, the homobifunctional cross-linking agent dimethyl suberimidate dihydrochloride (DMS) was used. The addition of DMS further augmented the positive surface charge of the DE particles, a phenomenon that has been demonstrated in previous studies [30,31,32]. Consequently, the resulting positively charged ADe-DMS complex captures negatively charged EVs by electrostatic bonding. The complete workflow of the DeSEI, which incorporates these ADe particles into a syringe–filter system, is shown in Figure 1B. In this process, ADe and DMS were first added to the cell culture medium (CCM). This step facilitates the electrostatic binding of EVs to the ADe particles. The mixture was then incubated for 10 min with gentle agitation. After incubation, the mixture was loaded into a syringe and passed through a syringe filter. The larger ADe-EV complexes were retained by the filter, while the rest of the medium passed through as filtrate. After washing the filter with phosphate-buffered saline (PBS) to remove unbound contaminants, the residual liquid was cleared by passing air through the syringe. To elute the captured EVs, the filter was treated with a high-pH elution buffer, which disrupts the electrostatic binding between the ADe and the EVs. This alkaline condition neutralizes the positive charge on the ADe surface by deprotonating the amine groups, which eliminates the electrostatic attraction. The resulting eluate, containing the purified EVs, was then collected for the downstream analysis. The entire DeSEI procedure is rapid, requiring only approximately 30 min to complete. The process is simple and cost-effective, as it does not require any specialized equipment. Thus, the DeSEI offers a streamlined and accessible alternative to conventional EV isolation methods, making it suitable for a wide range of research and clinical applications where purified EVs are required for a functional or molecular analysis.

### 2.2. The Optimization of the DeSEI Protocol

**Figure 2:**
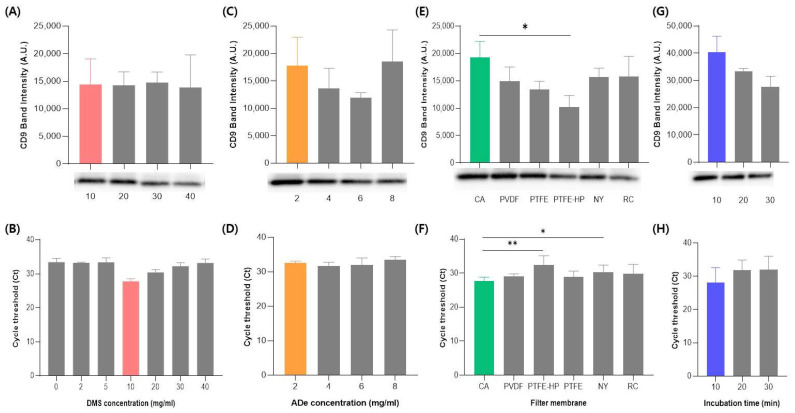
The optimization of the DeSEI protocol for EV isolation. The EV yield for each parameter was evaluated by quantifying the EV-specific protein marker CD9 via the Western blot analysis and the EV-enriched cargo miRNA-21 via the RT-qPCR. The optimization of the (**A**,**B**) DMS concentration, (**C**,**D**) ADe concentration, (**E**,**F**) syringe filter membrane type, and (**G**,**H**) sample incubation time. In all graphs, the selected optimal condition for each parameter is highlighted (DMS in red, ADe in orange, filter type in green, and incubation time in blue). The final optimized protocol was established with a DMS concentration of 10 mg/mL, an ADe concentration of 2 mg/mL, a cellulose acetate (CA) syringe filter, and a 10 min incubation time. Data are presented as mean ± standard deviation (*n* = 3). Significant differences are indicated as follows: * *p* < 0.05 and ** *p* < 0.01. Abbreviations: DeSEI, amine-functionalized Diatomaceous earth Syringe platform for EV Isolation; ADe, Amine-functionalized Diatomaceous earth; DMS, Dimethyl suberimidate dihydrochloride; and EV, extracellular vesicle.

To establish the optimal conditions for the EV isolation using the DeSEI, several key parameters were systematically evaluated. All experiments were performed on the same CCM sample, with the outcomes determined by the total protein concentration, Western blotting for the exosome marker CD9, and RT-qPCR analysis of microRNAs (miRNAs) targeting the miRNA-21. This set of indicators was deliberately chosen to provide a comprehensive evaluation of the yield, with CD9 representing a canonical vesicle surface protein (per MISEV guidelines) and miRNA-21 serving as an abundant and readily quantifiable internal miRNA marker. First, the concentration of the cross-linker, DMS, was optimized. Upon testing concentrations of 10, 20, 30, and 40 mg/mL, a concentration of 10 mg/mL yielded the highest EV recovery, as indicated by the most intense band in the Western blot analysis (Figure 2A). This finding was consistent with the total protein concentration results (Figure S1A). A similar trend was observed in the RT-qPCR analysis, which showed a notable decrease in efficiency at DMS concentrations below 10 mg/mL (Figure 2B). This may be attributed to excessive cross-linking, which could impede the release of EVs from the substrate or interfere with efficient lysis and RNA extraction. These findings suggest that the DMS concentration must be carefully optimized to balance the capture strength and downstream compatibility. Therefore, 10 mg/mL was selected as the optimal concentration to ensure a high efficiency while minimizing the reagent consumption. This concentration was used for all subsequent experiments. Next, the optimal amount of ADe was determined by testing 2, 4, 6, and 8 mg/mL. While the total protein concentration was slightly lower at 2 mg/mL compared to other conditions (Figure S1B), both the Western blotting (Figure 2C) and RT-qPCR (Figure 2D) demonstrated a comparable EV and miRNA recovery across all tested concentrations. Considering that excess, insoluble ADe particles could potentially clog the syringe filter pores without substantially increasing the yield of EVs, the lowest effective amount of 2 mg/mL was selected as optimal. The influence of different syringe filter membrane materials on the isolation efficiency was then investigated. Eight different membrane types were tested (CA, PVDF, PTFE, PTFE-HP, RC, NY, PES, and MCE). PES and MCE membranes were excluded from further analysis as they frequently tore upon contact with the ADe particles. Among the remaining six membranes, the cellulose acetate (CA) membrane demonstrated the highest recovery of both the Western blot (Figure 2E) and RT-qPCR (Figure 2F), respectively. Interestingly, it also yielded the lowest total protein concentration among the membranes tested (Figure S1C), which may suggest a higher purity of the isolated EV. Thus, the CA membrane was selected for the final protocol due to its superior performance in balancing the purity and yield. Finally, the incubation time for the ADe-EV binding step was optimized by comparing 10, 20, and 30 min intervals (Figure 2G,H and Figure S1D). The results revealed the highest efficiency at 10 min. Notably, while the total protein concentration remained similar across all time points (Figure S1D), the intensity of EVs’ marker bands on the Western blot decreased at 20 and 30 min (Figure 2G). This suggests that longer incubation times may lead to an increased non-specific binding of contaminating proteins, thereby reducing the relative purity of the isolated EVs. Therefore, a 10 min incubation was identified as optimal for achieving a high recovery and purity. Based on these experiments, the final, optimized DeSEI protocol was established with the following parameters: a DMS concentration of 10 mg/mL, an ADe amount of 2 mg/mL, a CA syringe filter membrane, and a reaction time of 10 min.

### 2.3. The Validation and Comparison of the DeSEI

**Figure 3:**
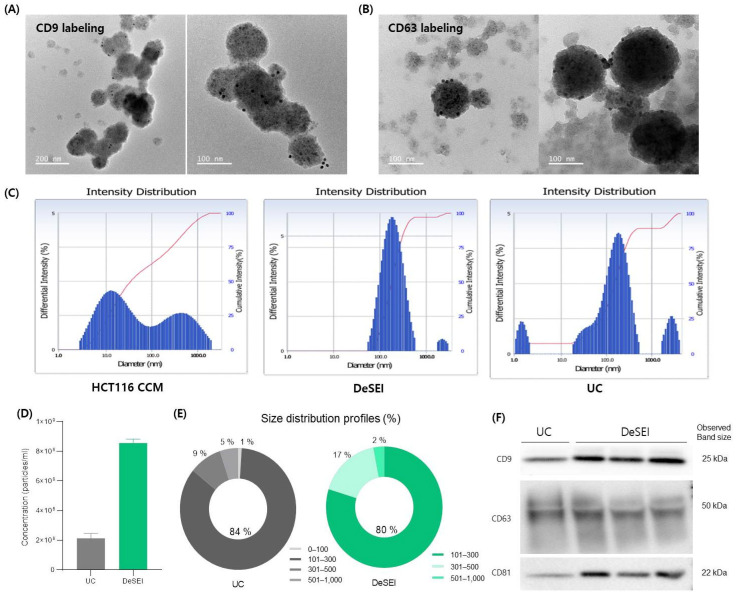
The physicochemical and molecular characterization of EVs isolated by the DeSEI. (**A**,**B**) The transmission electron microscopy (TEM) of isolated EVs. Representative images show spherical vesicles of approximately 100–200 nm, confirming their intact morphology. The immunogold labeling demonstrates the presence of the EV surface markers (**A**) CD9 and (**B**) CD63, indicated by attached gold nanoparticles (black dots). (**C**) The dynamic light scattering (DLS) analysis of the particle size distribution. While the initial HCT116 CCM (left) contains a heterogeneous particle population, EVs isolated by the DeSEI (center) show a distinct peak between 100 and 300 nm, a pattern comparable to that of EVs isolated by UC (right). (**D**,**E**) The nanoparticle tracking analysis (NTA) of isolated EVs. (**D**) The particle concentration and (**E**) size distribution profiles of EVs isolated using UC and DeSEI methods from the same starting sample volume. (**F**) The Western blot analysis of EV-specific protein markers (tetraspanins CD9, CD63, and CD81) in the DeSEI-isolated EVs. Abbreviations: DeSEI, amine-functionalized Diatomaceous earth Syringe platform for EV Isolation; CCM, cell culture media; UC, ultracentrifugation; and EVs, extracellular vesicles.

The quality, integrity, and physicochemical properties of EVs isolated by the DeSEI method were validated and compared against conventional techniques. The morphology and ultrastructure of the isolated particles were first examined. Transmission electron microscopy (TEM) images revealed intact, spherical vesicles, predominantly ranging from 100 to 200 nm in diameter (Figure 3A,B). To verify their identity as EVs, immunogold labeling confirmed the presence of canonical surface markers CD9 and CD63, visualized as distinct gold nanoparticles on the vesicle surfaces. These characteristics were highly comparable to those of EVs isolated by UC (Figure S2). These morphological features, including the spherical shape and gold nanoparticle labeling, are consistent with previous reports using immunogold TEM [38,39,40]. Complementing the imaging data, a dynamic light scattering (DLS) analysis profiled the particle size distribution. While the starting CCM showed a broad and heterogeneous particle profile, DeSEI-isolated samples exhibited a distinct, narrow peak centered within the 100–300 nm range (Figure 3C). This shift not only demonstrates the effective purification from the complex medium but also suggests a higher degree of sample homogeneity compared to UC. For the quantitative analysis, a nanoparticle tracking analysis (NTA) was performed. A direct comparison showed that the DeSEI yielded a higher particle concentration than the UC method from an equivalent sample volume (Figure 3D). The size distribution by the NTA was consistent with typical EV profiles and was highly similar to the UC control (Figure 3E), with comparable mean and mode particle sizes for both methods (Figure S3). Finally, the molecular signature of the isolated vesicles was confirmed by Western blotting. EVs isolated by the DeSEI were positive for the canonical EV markers CD9, CD63, and CD81 in Figure 3F, with notably higher expression levels compared to the UC-isolated positive controls, which is consistent with the increased particle concentration observed in Figure 3D. To assess the purity and composition of the sample, the isolated fractions were probed for various markers (Figure S4). The cellular contamination markers GM130 (Golgi) and calnexin (endoplasmic reticulum (ER)) were undetectable, indicating a high purity and a low cellular debris content. Additionally, the low or absent signal for the microvesicle marker ARF6 suggests that the isolated population is predominantly composed of tetraspanin-rich exosomes. Collectively, these data confirm that the DeSEI isolates a concentrated population of pure, intact EVs.

**Figure 4:**
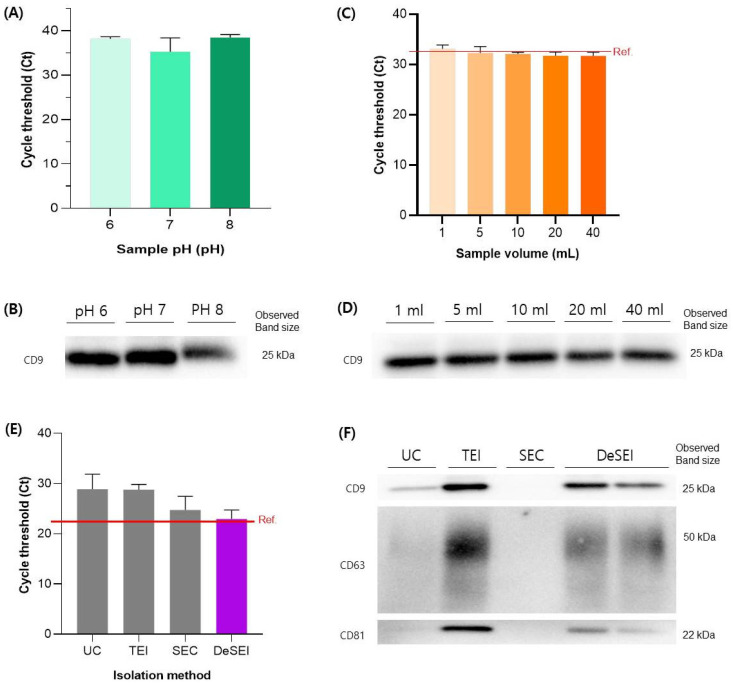
The performance and comparative efficiency of the DeSEI. (**A**,**B**) The evaluation of the EV isolation efficiency from samples with varying pH levels. (**A**) RT-qPCR analysis for miRNA-21, where darker green bars correspond to higher pH values (pH 6, 7, and 8). (**B**) Corresponding Western blot analysis for the CD9 marker. (**C**,**D**) A comparison of the EV isolation efficiency across varying sample volumes (1 mL to 40 mL). (**C**) RT-qPCR analysis for miRNA-21, where darker shades of orange correspond to larger sample volumes. (**D**) Corresponding Western blot analysis for the CD9 marker. (**E**,**F**) The evaluation of the DeSEI performance against other commercial EV isolation methods. (**E**) RT-qPCR analysis for miRNA-21, where the performance of DeSEI (purple bar) is compared against the other methods (grey bars); the red line indicates the reference value. (**F**) Corresponding Western blot analysis for the canonical EV markers CD9, CD63, and CD81. Abbreviations: DeSEI, amine-functionalized Diatomaceous earth Syringe platform for EV Isolation; EV, extracellular vesicle; UC, ultracentrifugation; SEC, size exclusion chromatography; and TEI, total exosome isolation kit.

To test the versatility of the platform, its performance was evaluated across different sample pH values and volumes. First, since the addition of the ADe and DMS results in a decrease in the pH of the sample, we investigated the effect of a varying pH on the isolation efficiency across a pH range of 6, 7, and 8. While the RT-qPCR for miRNA-21 (Figure 4A) and the total protein concentration (Figure S5A) remained stable across all conditions, the Western blotting for CD9 indicated an optimal performance at pH 6 and 7, with a slightly weaker signal at pH 8 (Figure 4B). This demonstrates that while the method is most efficient between pH 6 and 7, it remains effective at other pH levels, suggesting its applicability to diverse clinical samples. This trend is likely due to the deprotonation of amine groups on the ADe surface under alkaline conditions, which reduces the positive surface charge and weakens electrostatic interactions with negatively charged EV membranes. Next, to assess the scalability for the sample volume, we evaluated its performance across a range of volumes (1–40 mL), while keeping the total particle input constant at 10^8^ particles. The DeSEI successfully recovered EVs from all volumes tested (Figure 4 and Figure S5B). Importantly, both the miRNA (Figure 4C) and EV marker (Figure 4D) recovery showed no significant volume-dependent differences, confirming that the technology is scalable and performs reliably from small to large sample volumes. Moreover, its performance was evaluated across a panel of four biologically distinct cell lines. In addition to HCT116, EVs were isolated from a normal colon fibroblast line (CCD-18Co), a prostate cancer line (22Rv1), and a hepatic progenitor cell line (HepaRG). The results demonstrated a robust and consistent EV recovery from all cell types. The total protein concentration, as determined by the Bradford assay, was comparable for all four lines, ranging from 1.27 to 1.67 mg/mL (Figure S6A). Furthermore, the Western blot analysis of 25 µg of protein from each sample confirmed the strong presence of the canonical EV marker CD63 in all cases (Figure S6B). All isolated EV populations also exhibited the characteristic negative surface charge, with zeta potential values between −10 and −15 mV (Figure S6C). This negative surface charge is consistent with the previously reported zeta potential values for EVs [41,42,43]. Taken together, these results confirm that the DeSEI method effectively isolates EVs from diverse cellular origins, highlighting its broad applicability. The efficiency of the DeSEI was compared against three established methods: UC, a precipitation-based kit (Total Exosome Isolation, TEI), and SEC. For all comparisons, identical samples containing 10^8^ particles in 10 mL were used as the starting material. Regarding nucleic acid recovery, the DeSEI demonstrated a cycle threshold (Ct) value for miRNA-21 that most closely matched the reference value set by the direct extraction from 10^8^ particles (Figure 4E). These findings reveal that the DeSEI offers a vastly superior nucleic acid recovery, yielding approximately 62-fold, 58-fold, and 3.4-fold more material than the UC, TEI, and SEC methods, respectively. The analysis of protein markers by the Western blot revealed that while the TEI kit yielded the most intense bands, the DeSEI produced stronger signals for CD9, CD63, and CD81 than the UC method (Figure 4F), despite comparable total protein concentrations (Figure S7). In our results, the particle concentration obtained using the DeSEI was approximately 4-fold higher than that of UC (Figure 3D). Regarding EV protein markers (Figure 3F), CD9 and CD81 exhibited 1.5- to 1.7-fold higher signal intensities in the DeSEI, while CD63 was slightly lower (0.89-fold). Thus, while particle and protein marker-based measurements showed moderate increases, the substantially higher RNA recovery highlights the effectiveness of the DeSEI in preserving the molecular cargo, particularly for downstream transcriptomic analyses. These findings position the DeSEI as a method with a balanced performance, offering a superior nucleic acid recovery and a highly competitive protein marker yield compared to other widely used techniques.

### 2.4. One-Step DeSEI Protocol for Direct EV-Derived miRNA Extraction

**Figure 5:**
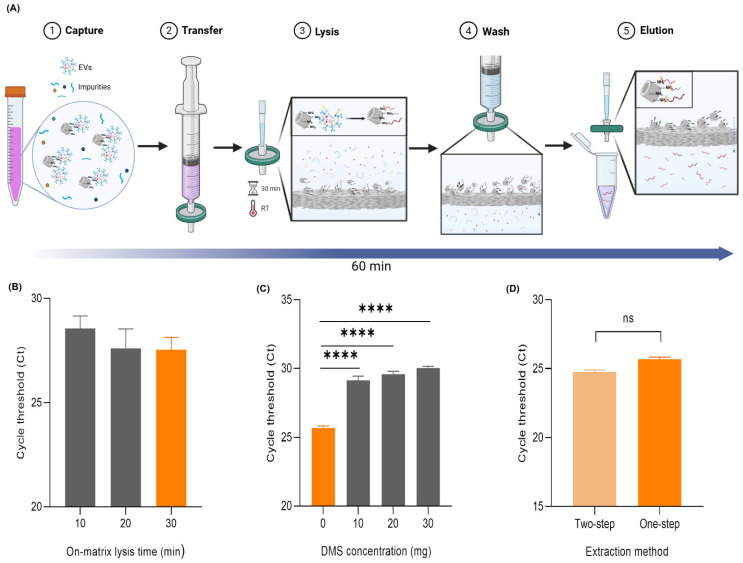
The performance and comparative efficiency of the DeSEI. (**A**) The workflow of the one-step miRNA extraction using the DeSEI. The initial EV capture and transfer steps are performed as previously described. Subsequently, in the lysis step, a lysis buffer is added, and the sample is incubated for 30 min. A wash step is then performed to remove residual debris. Finally, an elution buffer is added to collect the purified miRNA. The entire five-stage process is completed in under 60 min. (This figure was created with BioRender.com). (**B**) The optimization of the lysis incubation time, with the optimal duration of 30 min highlighted in orange. (**C**) The optimization of the additional DMS concentration in the lysis buffer. The highest miRNA yield was highlighted in orange. (**D**) A comparison of the miRNA extraction efficiency between the integrated one-step DeSEI method and a conventional two-step method, where the DeSEI isolation is performed prior to the miRNA extraction using a commercial kit. Significant differences are indicated as follows: **** *p* < 0.0001, ns (not significant) *p* > 0.05. For all optimization and comparison experiments, the extraction efficiency was quantified by measuring miRNA-21 levels via a RT-qPCR. Data are presented as the mean ± standard deviation (*n* = 3). Abbreviations: DeSEI, amine-functionalized Diatomaceous earth Syringe platform for EV Isolation; EVs, extracellular vesicles.

The development of a one-step miRNA extraction protocol was inspired by prior research on the DeSEI. A previous study demonstrated that the cross-linker DMS can form stable amidine bonds between the primary amine groups of nucleic acids and those on the ADe surface [31,32]. We hypothesized that this principle could be adapted to create a direct extraction method for EV-derived miRNA, thereby streamlining its process. The conventional process for extracting EV-derived miRNA typically involves the use of commercial miRNA extraction kits following the EV isolation, which is time-consuming and involves multiple complex steps. To simplify the complex process, a one-step workflow was designed using the DeSEI platform for the direct extraction of EV-derived miRNA (Figure 5A). The initial steps, where EVs are captured on the ADe, are identical to the standard EV isolation protocol. This is followed by an on-filter lysis step. A lysis buffer containing NP-40 is introduced to saturate the filter, and a 30 min incubation at room temperature disrupts the EV membranes. This releases the internal miRNA cargo, which is then captured by the ADe via the pre-existing DMS cross-linkages. Following lysis, a PBS wash removes unbound materials, and a high-pH buffer is used to selectively elute the purified miRNA. This integrated process is completed in approximately 60 min using only a syringe and a filter, eliminating the need for additional specialized equipment. The one-step EV-derived miRNA extraction protocol was first established through a series of optimization experiments. The optimization of the on-matrix lysis time revealed that while shorter periods were insufficient for a complete miRNA release, a 30 min incubation yielded the most stable and efficient recovery and was therefore adopted (Figure 5B). Next, we evaluated the effect of the amount of DMS added to the lysis buffer. Contrary to the hypothesis that more DMS would enhance the capture, adding extra cross-linkers did not improve efficiency (Figure 5C), suggesting the initial amount of DMS used for the EV capture is sufficient for the subsequent miRNA binding. Finally, the performance of the optimized one-step protocol was validated against a conventional two-step process (i.e., DeSEI EV isolation followed by a commercial kit-based miRNA extraction). Although the two-step method was marginally more efficient, with the average Ct values for miRNA-21 differing by only approximately one cycle (24.75 vs. 25.68, Figure 5D), this difference was not statistically significant, indicating that both approaches exhibit a similar efficiency. To further assess the RNA selectivity, additional miRNAs (U6, hsa-let-7c, hsa-miR-7, hsa-miR-124, and hsa-miR-423) were tested and successfully extracted using both the one-step and conventional two-step protocols (Figure S8). No statistically significant differences were observed in the Ct values between the two methods across all tested miRNAs, indicating a consistent extract efficiency regardless of the miRNA identity. Therefore, despite a marginal difference in yield, the one-step DeSEI protocol represents a powerful and viable alternative, dramatically reducing the time and equipment required for the EV-derived miRNA analysis and further highlighting the versatility of the platform.

### 2.5. User-Friendly Cartridge System (I-PULL) with DeSEI

**Figure 6:**
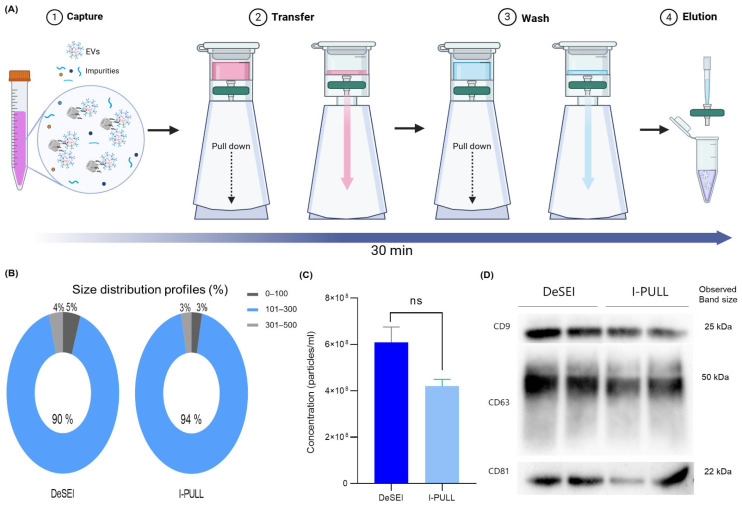
The schematic and characterization of the I-PULL cartridge with the DeSEI. (**A**) The I-PULL device for a simplified DeSEI workflow. While the fundamental method is unchanged, the I-PULL cartridge simplifies the process. It features an integrated syringe filter and operates on a vacuum-assisted principle. Loading the sample and pulling down the lower section creates negative pressure, which forces the solution through the filter for rapid separation. (This figure was created with BioRender.com). (**B**,**C**) The nanoparticle tracking analysis (NTA) of isolated EVs. (**B**) Size distribution profiles and (**C**) the particle concentration of EVs isolated using the DeSEI and I-PULL methods from the same starting sample volume. Significant differences are indicated as follows: ns (not significant) *p* > 0.05. (**D**) The Western blot analysis of EV-specific protein markers (tetraspanins CD9, CD63, and CD81) in the DeSEI-isolated and I-PULL-isolated EVs. Abbreviations: DeSEI, amine-functionalized Diatomaceous earth Syringe platform for EV Isolation; EVs, extracellular vesicles.

To enhance the user-friendliness and versatility of the DeSEI, an alternative workflow was used using a custom-designed cartridge, termed ‘I-PULL’ (INFUSIONTECH, Republic of Korea) [33]. Figure 6A illustrates the operational schematic of this device. While the fundamental biochemical principles of the EV capture are identical to the standard syringe method, the I-PULL modifies the physical mechanism for passing solutions through the filter. The process begins by loading the sample mixture, containing the ADe-DMS-EV complexes, into the top of the cartridge. After the lid is closed, pulling the lower body downwards generates a vacuum, which draws the solution through the integrated filter. This same vacuum-assisted procedure is repeated for the subsequent wash step. For the final step, the syringe filter is detached from the cartridge, and the purified EVs are collected by applying the elution buffer directly to the syringe filter. The total processing time for this cartridge-based method remains equivalent to that of the standard syringe technique. The efficacy of the I-PULL cartridge was evaluated by a direct comparison with the DeSEI (original syringe-based protocol). The NTA showed a strong concordance between the two methods. The mean and mode particle sizes were nearly identical (Figure S9A,B), with approximately 90% of particles falling within the 100–300 nm range (Figure 6B and Figure S9C). Although the particle concentration by the I-PULL was slightly lower, the difference was not statistically significant, indicating comparable isolation efficiencies between the two methods (Figure 6C). Next, these were corroborated by the Western blot analysis for the EV markers CD9, CD63, and CD81 (Figure 6D). Both the I-PULL and DeSEI resulted in the clear detection of all EV protein markers, with only minor variations in the band intensity observed between the two. This successful adaptation confirms that the core DeSEI technology is highly versatile and not limited to a single format, opening possibilities for its integration into various user-friendly devices for a simplified EV isolation.

## 3. Discussion

This study introduces the DeSEI, a rapid and simple technology developed to address the persistent limitations of conventional EV isolation methods. By employing DE within an SPE framework, our platform enables equipment-free EV isolation. Through systematic optimization and thorough characterization, we have established a protocol that ensures the recovery of pure, intact EVs. A key advantage of the DeSEI is its operational robustness. Our results demonstrated a consistent performance across wide ranges of pH and sample volumes, addressing critical constraints of established methods, such as the volume limitations of SEC or the poor yields of UC with small samples. In direct comparisons, the DeSEI demonstrated a highly competitive performance. The efficiency of the platform was particularly pronounced in the miRNA recovery. In this aspect, the recovery rate is nearly 60-fold higher than that of UC or the precipitation method (TEI). With its procedural simplicity, rapid processing time (~30 min), and equipment-free operation, the DeSEI represents a promising alternative to conventional technologies for EV research. The versatility of the DeSEI extends beyond simple EV isolation, as demonstrated by two novel applications. First, we established a one-step method for the direct extraction of EV-derived miRNA, which drastically reduces the time and complexity of conventional complicated procedures. This innovation holds significant potential for accelerating biomarker research. Second, the technology was successfully adapted into a user-friendly cartridge format, the “I-PULL”, which achieved a performance comparable to the standard syringe method. Through these applications, the DeSEI demonstrates its considerable adaptability.

Despite these promising results, the current DeSEI protocol has limitations that warrant discussion. The primary constraint is its reliance on electrostatic interactions, which can lead to the co-isolation of non-EV proteins and other contaminants, an issue that may be more pronounced with complex clinical samples. Indeed, some optimization experiments yielded high total protein concentrations but relatively weak Western blot signals for EV markers, a discrepancy we attribute to non-specific protein binding. While the protocol was optimized for conditions that maximized the EV marker recovery, some non-specific binding could not be fully eliminated. Therefore, further studies and additional optimization are required to validate its reliable application to diverse and complex clinical samples. Future work will focus on mitigating this non-specific binding, potentially through modifications to the wash and elution buffers, such as adjusting their ionic strength or pH to disrupt weaker, non-specific interactions. It is known that the EV surface charge is heterogeneous, influenced by factors such as the lipid composition (e.g., surface exposure of phosphatidylserine) and sialic acid density. To assess the generalizability of the DeSEI platform and the potential influence of the EV surface charge heterogeneity, we confirmed its robust performance across four distinct cell lines. While this could theoretically lead to a preferential capture of more negatively charged EVs, we did not observe a strong correlation between the subtle zeta potential differences and recovery yields in our study. This suggests that the DeSEI is effective across a common range of EV surface charges, although a more detailed investigation to precisely quantify this relationship remains a valuable future direction. In particular, as extracellular vesicles encompass heterogeneous subtypes, including exosomes and microvesicles, future studies should explore whether the DeSEI capture efficiency varies across these subpopulations. The robustness of our method was confirmed by its consistent performance under various conditions, with its high reproducibility verified through triplicate experiments. However, as these validations were performed exclusively using the cell culture medium, the immediate priority for future work is the extensive validation of the platform using clinical samples. This will be essential for assessing its performance and potential for translation into diagnostic settings.

The DeSEI is a robust and versatile tool that overcomes many practical limitations of conventional methods. While its reliance on electrostatic interactions presents a clear avenue for future refinement, this technology holds significant promise for a wide range of applications where rapid EV isolation and direct miRNA extraction are critical.

## 4. Materials and Methods

### 4.1. Cell Culture and Media Collection

The HCT116 colorectal cancer cell line was obtained from the Korean Cell Line Bank (KCLB, Cat no. 10247, Seoul, Republic of Korea) and maintained in Dulbecco’s Modified Eagle Medium (DMEM, Thermo Fisher Scientific, Waltham, MA, USA) supplemented with 1% Antibiotic–Antimycotic (DAWINBIO, Seoul, South Korea) and 10% exosome-depleted Fetal Bovine Serum (FBS, Thermo Fisher Scientific). The cells were cultured in a humidified incubator at 37 °C with a 5% CO_2_ atmosphere. CCM was collected when cells reached approximately 80% confluence (typically after 72 h), yielding about 10 mL per plate. The collected CCM was either used immediately for experiments or stored at −20 °C for up to 4 weeks or at −80 °C for long-term storage. The HCT116 cell line was chosen as the model system for this study due to its rapid proliferation, high EV-secreting capacity, phenotypic stability ensuring experimental reproducibility, and its extensive characterization in existing EV literature [41,44]. To validate the applicability of the DeSEI method across different cell types, we included three additional cell lines: CCD-18Co (normal fibroblast), 22Rv1 (prostate cancer), and HepaRG (hepatic-like). All cells were cultured under standard conditions using EV-depleted FBS during the EV collection phase.

### 4.2. Synthesis of Amine-Functionalized Diatomaceous Earth (ADe)

ADe was synthesized based on a previously reported protocol [30,31,32,33,45]. Briefly, 0.5 g of DE (Cat no. 56678, Sigma-Aldrich, St. Louis, MO, USA) was washed extensively with distilled water (DW) and filtered through a sieve to isolate particles smaller than 20 μm. In a separate container, 2 mL of APDMS (Cat no. 371890, Sigma-Aldrich) was added dropwise to 100 mL of 95% ethanol previously acidified with acetic acid to pH 5.0. The prepared DE particles were then resuspended in this APDMS–ethanol mixture. The suspension was incubated for 4 h at room temperature with vigorous agitation. After incubation, the resulting ADe particles were collected, washed sequentially with ethanol and DW, and dried. The dried ADe was stored at room temperature until use.

### 4.3. EV Isolation and EV-Derived miRNA Extraction Using DeSEI

For EV isolation, the collected CCM was mixed with ADe and DMS (Cat no. 179523, Sigma-Aldrich), and the mixture was rotated for 10 min. The solution was then slowly loaded into a syringe filter (CA, 25 mm, 0.45 μm, GVS S.p.A., Bologna, Italy) using a syringe. A washing step with 2 mL of PBS was performed to remove residual DMS and avoid potential interference in downstream analyses. Subsequently, residual liquid was removed by passing air through the syringe filter. Finally, 300 μL of elution buffer (10 mM NaHCO_3_, pH ~10.6) or 300 μL of Radioimmunoprecipitation assay (RIPA) buffer was added to the filter and incubated for 10 min. The final eluate was then collected for analysis. For EV-derived miRNA extraction, the procedure was identical up to the sample loading step. After loading, a lysis solution using NP-40 (Cat no. J60766, Thermo Fisher Scientific) was introduced into the syringe until the filter was saturated. The filter was then incubated for 30 min at room temperature. Following incubation, the filter was washed with PBS, and 300 μL of elution buffer was added to saturate the filter again. After a 10 min incubation, the eluate containing the miRNA was collected in a new tube. All eluted samples (EVs, proteins, or EV-derived miRNAs) were either used immediately or stored at −80 °C.

### 4.4. EV Isolation Using Conventional Methods

For comparison, EVs were also isolated using UC, a commercial precipitation kit (TEI), and SEC.

UC: Samples underwent sequential centrifugation at 300× *g* for 10 min, 2000× *g* for 20 min, and 10,000× *g* for 30 min to remove cells and debris. The supernatant was then ultracentrifuged at 110,000× *g* for 70 min using an SW41 Ti rotor (Beckman Coulter, Brea, CA, USA). After discarding the supernatant, the pellet was washed with PBS and subjected to a second ultracentrifugation step at 110,000× *g* for 70 min. The final pellet containing EVs was resuspended in 300 µL of 1× PBS.

Precipitation Kit (TEI): EVs were isolated using the commercial kit, TEI (Total Exosome Isolation Reagent (from cell culture media), Cat no. 44778359, Invitrogen, Carlsbad, CA, USA) according to the manufacturer’s protocol.

SEC: SEC was performed using qEVoriginal/35 nm columns (IZON, Christchurch, New Zealand) following the manufacturer’s instructions. Five fractions of 400 µL each were collected, and the second fraction was used for downstream analysis.

### 4.5. Characterization of Isolated EVs

Isolated EVs were characterized using various techniques to confirm their morphology, size distribution, concentration, and protein marker expression.

TEM: The morphology of isolated EVs was examined by TEM. EV samples were immobilized on formvar/carbon-coated copper grids. For immunogold labeling, the grids were incubated with primary antibodies incubated with the following EV marker primary antibodies: anti-CD9 (Cat no. ab236630, Abcam, Cambridge, UK) and anti-CD63 (Cat no. ab193349, Abcam). Following the primary antibody incubation and subsequent washes, the grids were incubated with the appropriate secondary antibodies: 10 nm gold-conjugated goat anti-mouse IgG (Cat no. ab39619, Abcam) and 10 nm gold-conjugated donkey anti-rabbit IgG (Cat no. ab39597, Abcam). The grids were observed under a TEM (JEM-F200, JEOL, Tokyo, Japan).

NTA: The size distribution and concentration of isolated EVs were measured by NTA using a NanoSight NS300 (Malvern Panalytical, Malvern, UK). Samples were diluted in PBS to an appropriate concentration, and three videos of 60 s each were recorded and analyzed by the NTA software to determine the mean particle size and concentration.

DLS: The hydrodynamic diameter of particles was measured by DLS using an ELS-Z1000 instrument (Otsuka Electronics, Osaka, Japan). The analysis was performed on EVs isolated by DeSEI, UC, and the CCM before the EV isolation process to assess capture efficiency.

Western blot analysis: The presence of EVs marker proteins (e.g., CD9, CD63, CD81) and non-EVs marker (e.g., Calnexin, GM130, ARF3) was confirmed by Western blotting. EVs were lysed in RIPA buffer, and the total protein concentration was determined using the Bradford assay. Equal amounts of protein (25 µg per lane) were loaded onto a 10% SDS-PAGE gel and subsequently transferred to a PVDF membrane using a Bio-Rad Mini Trans-Blot cell. The membrane was blocked with 5% non-fat skim milk in Tris-buffered saline with 0.1% Tween 20 (TBS-T) for 1 h at room temperature. The blot was then incubated overnight at 4 °C with the following primary antibodies: anti-CD9 (Cat no. ab236630), anti-CD63 (Cat no. ab193349, Abcam), anti-CD81 (Cat no. ab79559, Abcam), anti-ARF6 (Abcam, ab131261), anti-Calnexin (Cat no. 2679S, Cell Signaling Technology, Danvers, MA, USA), and anti-GM130 (Cat no. ab52649, Abcam). After washing, the membrane was incubated for 1 h at room temperature with one of the following HRP-conjugated secondary antibodies: polyclonal goat anti-mouse IgG (Cat no. ab6789, Abcam) or goat anti-rabbit IgG (Cat no. ab205718, Abcam). Immunoreactive bands were visualized using a chemiluminescent substrate (ECL solution, Bio-Rad, Hercules, CA, USA), and signals were captured with a molecular imager (ChemiDoc XRS+, Bio-Rad). Band intensity was analyzed using ImageJ (version 1.54k, National Institutes of Health (NIH), Bethesda, MD, USA).

Zeta potential: Zeta potential measurements were performed to determine the surface charge of the isolated EVs. For analysis, samples were diluted in PBS. All measurements were conducted using a Zetasizer Lab instrument (Malvern Instruments, Malvern, UK).

Statistical analysis: Group differences and method comparisons were assessed using two-tailed Student′s *t*-tests or Mann–Whitney tests, depending on whether the data were parametric or non-parametric. For analyses involving more than two groups, a one-way Kruskal–Wallis ANOVA was performed, followed by a Dunn′s multiple comparisons test for post hoc analysis. All analyses and visualizations were performed using GraphPad Prism 9 (GraphPad Software, La Jolla, CA, USA).

### 4.6. EV-Derived miRNA Extraction and RT-qPCR

EV-derived miRNA was extracted using the Total Exosome RNA and Protein Isolation Kit (Invitrogen) according to a modified protocol. Briefly, 200 µL of PBS was added to the EV-bound substrate, followed by the addition of 200 µL of denaturing solution (preheated to 37 °C). The mixture was incubated on ice for 5 min. The solution was then collected, mixed with 400 µL of Acid–Phenol:Chloroform, and vortexed for 60 s. The mixture was centrifuged for 5 min at 13,000× *g* at room temperature to separate the aqueous and organic phases. The upper aqueous phase was transferred to a fresh tube and mixed with 1/3 volume of 100% ethanol. This mixture was passed through a filter cartridge by centrifugation at 13,000× *g* for 30 s. The flow-through, containing small RNA, was collected and mixed with 1/3 volume of 100% ethanol. This new mixture was passed through the same filter cartridge. The filter was then washed sequentially with 700 µL of Wash Solution 1 and twice with 500 µL of Wash Solution 2. After drying the filter by centrifugation for 1 min at 13,000× *g*, the filter was placed in a new collection tube. Finally, the small RNA was eluted twice by adding 50 µL of preheated (95 °C) nuclease-free water to the filter and centrifuging for 30 s at 13,000× *g*. The final eluate (~100 μL) was stored at −20 °C.

For reverse transcription (RT), an RT Master Mix was prepared for each sample using the MicroRNA Reverse Transcription Kit (Applied Biosystems, Foster City, CA, USA) and specific stem-loop RT primers for the target miRNA. The master mix was added to PCR tubes containing 3 µL of the extracted RNA. The tubes were briefly centrifuged and incubated on ice for 5 min. The RT reaction was performed in a thermocycler with the following program: 16 °C for 30 min, 42 °C for 30 min, and 85 °C for 5 min, followed by a hold at 4 °C. For qPCR, 5 µL of the resulting cDNA was used as a template in a 20 µL reaction volume containing 10 µL of AccuPower 2× GreenStar qPCR Master Mix and 2.5 µM of each forward and reverse primer. The qPCR was performed on a QuantStudio 3 Real-Time PCR Instrument (Thermo Fisher Scientific) with the following cycling conditions: an initial denaturation at 95 °C for 10 min, followed by 40 cycles of 95 °C for 10 s, 60 °C for 20 s, and 72 °C for 20 s. The primer sequences can be found in Supporting Information Table S1.

## 5. Conclusions

In summary, the DeSEI technology provides a simple, rapid, and equipment-free solution for the high-efficiency isolation of EVs. Our findings demonstrate that its performance is not only comparable to gold-standard methods but, in key applications like nucleic acid recovery, is significantly superior. Its confirmed adaptability for diverse applications, from basic research to point-of-care systems, firmly establishes its potential to accelerate the progress of EV-based science and medicine significantly.

## Data Availability

The original contributions presented in this study are included in the article/Supplementary Material. Further inquiries can be directed to the corresponding author.

## Supplementary Materials

The following supporting information can be downloaded at https://www.mdpi.com/article/10.3390/ijms26146843/s1.

## Abbreviations

The following abbreviations are used in this manuscript:

DeSEI	Amine-functionalized Diatomaceous earth Syringe platform for EV Isolation
DE	Diatomaceous earth
ADe	Amine-functionalized Diatomaceous earth
EVs	Extracellular vesicles
UC	Ultracentrifugation
TEI	Total exosome isolation kit
SEC	Size exclusion chromatography
SPE	Solid-phase extraction
TEM	Transmission electron microscopy
DLS	Dynamic light scattering
NTA	Nanoparticle tracking analysis
miRNAs	MicroRNAs
Ct	Cycle threshold
CCM	Cell culture media
APDMS	3-Aminopropyl(diethoxy)methylsilane
DMS	Dimethyl suberimidate dihydrochloride
RIPA	Radioimmunoprecipitation assay
TBS	Tris-buffered saline
PBS	Phosphate-buffered saline
DMEM	Dulbecco’s modified eagle medium
FBS	Fetal bovine serum
DW	Distilled water
