# Supplementary material for: Amine-Modified Diatomaceous Earth Syringe Platform (DeSEI) for Efficient and Cost-Effective EV Isolation
Source: Int J Mol Sci. 2025 Jul 16;26(14):6843. doi: 10.3390/ijms26146843 (PMC12295286; doi:10.3390/ijms26146843)
Supplement: Supplementary file 1 [file ijms-26-06843-s001.zip › ijms-3740269-supplementary.pdf]

Amine-Modified Diatomaceous Earth Syringe Platform (DeSEI) for Efficient and Cost-Effective EV Isolation

Hyo Joo Lee[a], Jinkwan Lee[b], Namheon Kim[b], and Yong Shin[a,*]

[a]*Department of Biotechnology, College of Life Science and Biotechnology, Yonsei University, Seoul 03722, Republic of Korea*

[b]*INFUSIONTECH, 38 Heungan-daero, 427 Beon-gil, Dongan-gu, Anyang-si 14059, Republic of Korea*

*Corresponding authors: Email: shinyongno1@yonsei.ac.kr (Y. Shin)

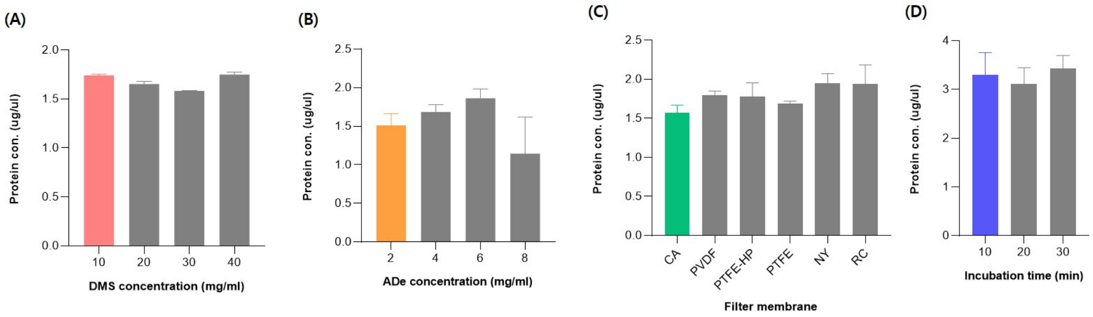

Figure S1. Protein concentration results of optimization experiments for EVs Isolation using DeSEI. (A) Optimization experiment for the concentration of DMS used as a cross-linking agent. (B) We conducted an optimization experiment for the concentration of ADe. (C) Membrane types optimization experiment of syringe filters. (D) Incubation time after ADe and DMS adding to the sample. Highlighted bars in each graph indicate the best performing conditions among the tested conditions. Across all experiments, data are presented as mean ± standard deviation (n = 3). Abbreviations: DeSEI, amine-functionalized Diatomaceous earth Syringe platform for EV Isolation; ADe, Amine-functionalized Diatomaceous earth; DMS, Dimethyl suberimidate dihydrochloride; EV, extracellular vesicle.

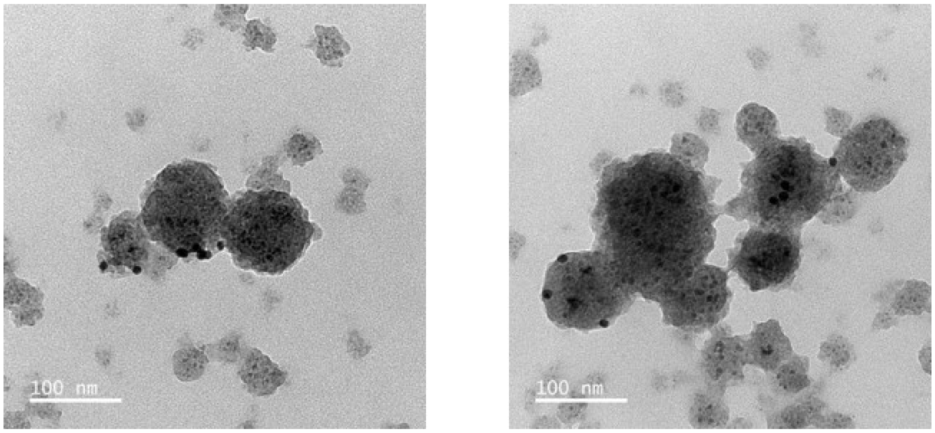

Figure S2. TEM micrograph showing a UC-isolated EV with CD63 immunogold labeling. Abbreviations: TEM, Transmission electron microscopy; EV, extracellular vesicle.

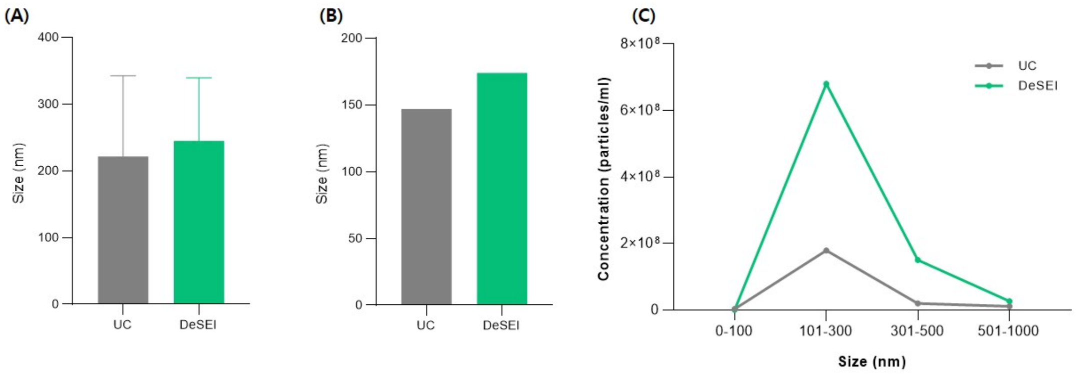

Figure S3. Nanoparticle tracking analysis (NTA) of EVs isolated by DeSEI and UC. (A) Mean and (B) mode particle sizes, **(C)** particle size distribution profile, plotted as concentration versus size, of EVs isolated using DeSEI (green) and UC (gray) methods. Abbreviations: DeSEI, amine-functionalized Diatomaceous earth Syringe platform for EV Isolation; UC, Ultracentrifugation; EV, extracellular vesicle.

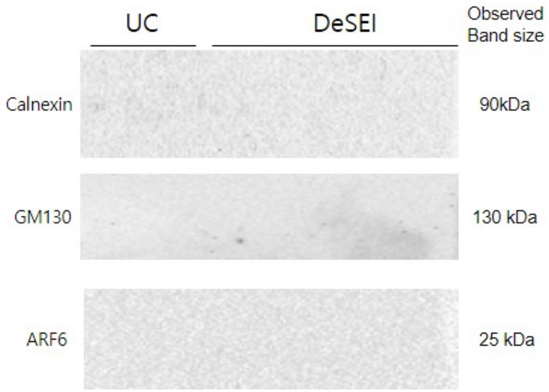

Figure S4. Assessment of Purity and Composition of Isolated EVs. Western blot analysis to assess microvesicle and cellular contamination in DeSEI-isolated EVs and UC controls: ARF6 (microvesicle), GM130 (Golgi), and Calnexin (Endoplasmic reticulum). Abbreviations: DeSEI, amine-functionalized Diatomaceous earth Syringe platform for EV Isolation; UC, Ultracentrifugation; EV, extracellular vesicle

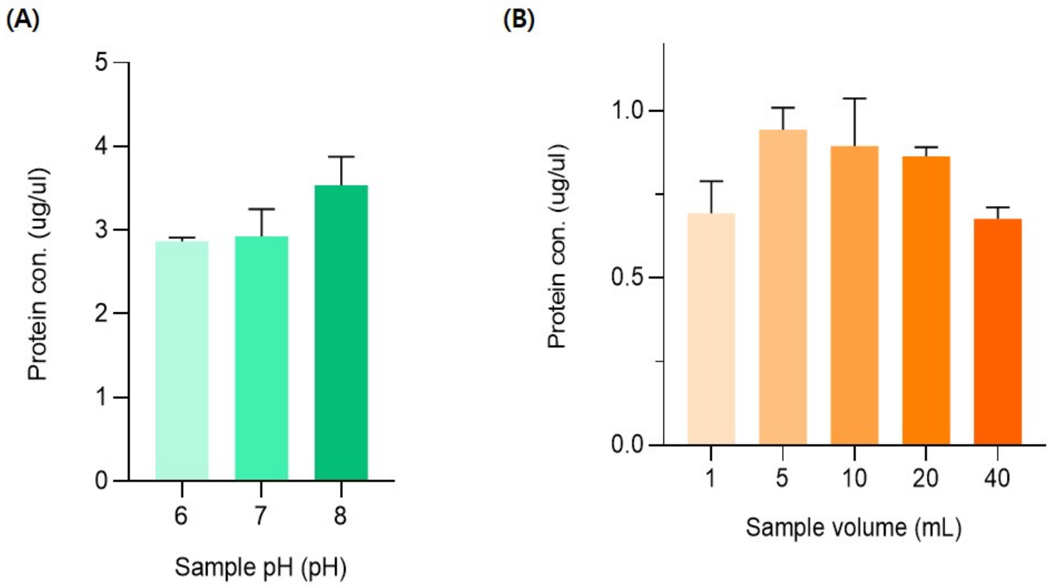

Figure S5. Protein concentration results from efficiency comparison experiments. (A) Protein concentration from samples with varying pH levels. (B) Protein concentration from different starting sample volumes.

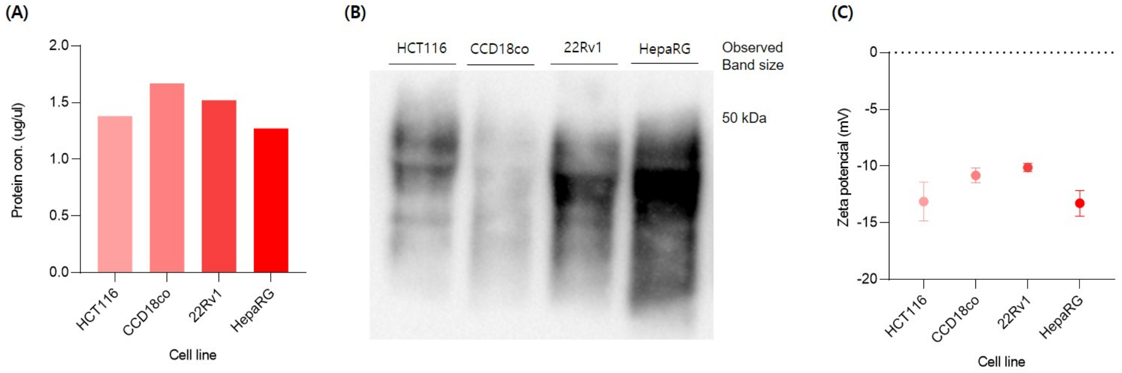

Figure S6. Versatility of the DeSEI Method for EV Isolation from Diverse Cell Lines. EVs were isolated using DeSEI from four different cell lines: HCT116, CCD18co, 22Rv1, and HepaRG. The isolated EVs were then characterized. (A) Total protein concentration of the isolated EV, quantified by the Bradford assay. (B) Western blot analysis confirming the presence of canonical EV markers (CD63). (C) Zeta potential measurements for each isolated EV population

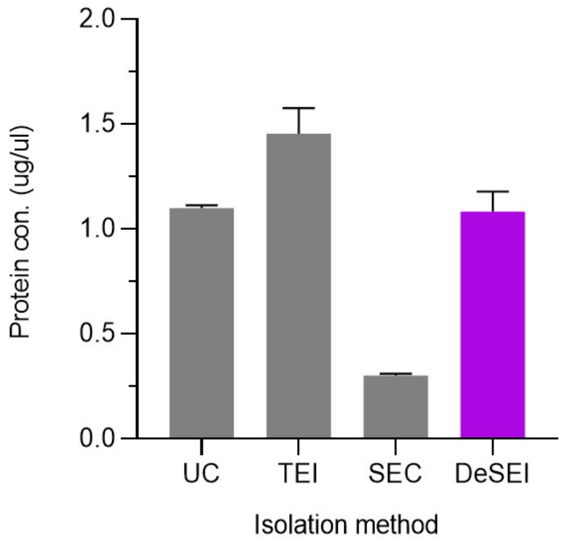

Figure S7. Protein concentration from various isolation methods. Abbreviations: DeSEI, amine-functionalized Diatomaceous earth Syringe platform for EV Isolation; UC, Ultracentrifugation; SEC, size exclusion chromatography; TEI, Total exosome isolation kit.

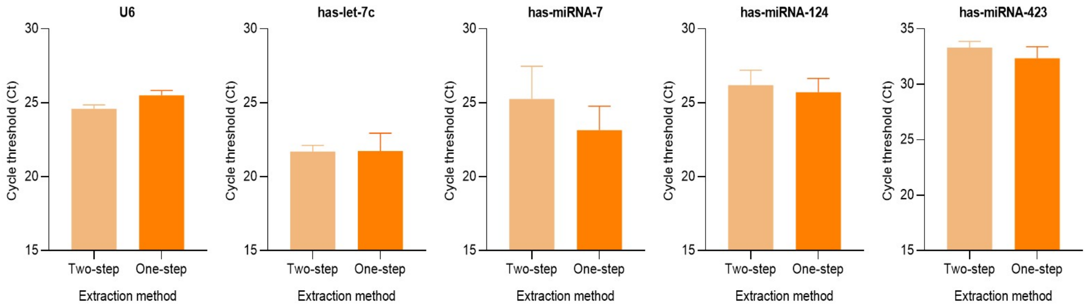

Figure S8. Efficacy of the One-Step DeSEI Method for miRNA Extraction. The performance of the one-step protocol was compared to a conventional two-step method of U6, hsa-let-7c, hsa-miRNA-7, hsa-miRNA-124, and hsa-miRNA-423 via RT-qPCR. Values represent the mean ± SD from three replicates.

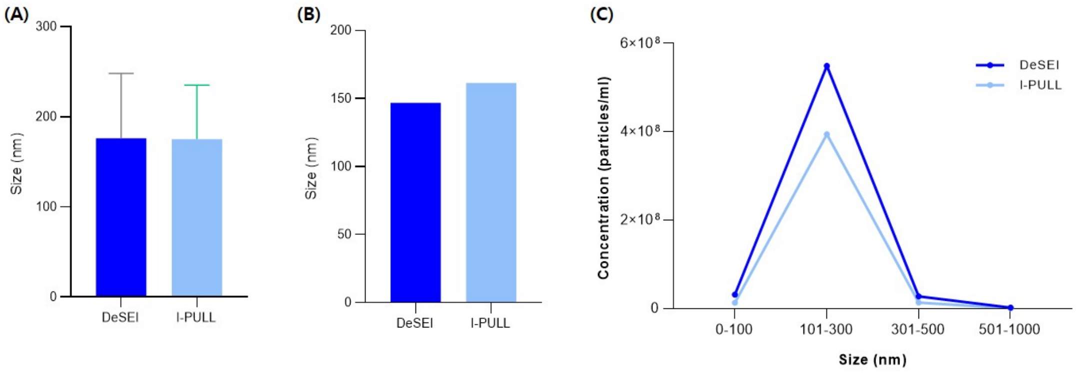

Figure S9. Nanoparticle tracking analysis (NTA) of EVs isolated by DeSEI and I-PULL. (A) Mean and (B) mode particle sizes, and **(C)** particle size distribution profile, plotted as concentration versus size, of EVs isolated using DeSEI (blue) and I-PULL (light blue). Abbreviations: DeSEI, amine-functionalized Diatomaceous earth Syringe platform for EV Isolation; EV, extracellular vesicl

Table S1. Primer Sequences for miRNA Amplification.

**Primer**	**Sequences (5’ - 3’)**
Stem loop primer hsa-miRNA-21	GTCGTATCCAGTGCAGGGTCCGAGGTATTCGCACTGGATACGACTCAACA
hsa-miRNA-21 Forward	GCCCGCTAGCTTATCAGACTGATG
hsa-miRNA-21 Reverse	CAGTGCAGGGTCCGAGGT
Stem loop primer U6	CGCTTCACGAATTTGCGTGTCAT
U6 Forward	CTCGCTTCGGCAGCACA
U6 Reverse	AACGCTTCACGAATTTGCGT
Stem loop primer hsa-let-7c	GCAUCCGGGUUGAGGUAGUAGGUUGUAUGGUUUAGAGUUACACCCUGGGAGUUAACUGUACAACCUUCUAGCUUUCCUUGGAGC
hsa-let-7c Forward	ACACTCCAGCTGGGTGAGGTAGTAGGTTGT
hsa-let-7c Reverse	TGGTGTCGTGGAGTCG
Stem loop primer hsa-miRNA-7	UUGGAUGUUGGCCUAGUUCUGUGUGGAAGACUAGUGAUUUUGUUGUUUUUAGAUAACUAAAUCGACAACAAAUCACAGUCUGCCAUAUGGCACAGGCCAUGCCUCUACAG
hsa-miRNA-7 Forward	TGGAAGACTAGTGATTTTG
Stem loop primer hsa-miRNA-124	AGGCCUCUCUCUCCGUGUUCACAGCGGACCUUGAUUUAAAUGUCCAUACAAUUAAGGCACGCGGUGAAUGCCAAGAAUGGGGCUG
hsa-miRNA-124 Forward	TTCACAGCGGACCTTGA
Stem loop primer hsa-miRNA-423	AUAAAGGAAGUUAGGCUGAGGGGCAGAGAGCGAGACUUUUCUAUUUUCCAAAAGCUCGGUCUGAGGCCCCUCAGUCUUGCUUCCUAACCCGCGC
hsa-miRNA-423 Forward	GGGCAGAGAGCGAGAC
Universal Reverse	GAACATGTCTGCGTATCTC
